# All in the Name of Artificial Intelligence: A Commentary on Linardon (2025)

**DOI:** 10.1002/eat.24446

**Published:** 2025-04-15

**Authors:** Pia Burger, Sreejita Ghosh

**Affiliations:** ^1^ GGz Eindhoven Eindhoven the Netherlands; ^2^ Department of Pediatrics Emma Children's Hospital, Amsterdam UMC Amsterdam the Netherlands; ^3^ GGz Oost‐Brabant Boekel the Netherlands; ^4^ Department of Mathematics and Computer Science Technical University of Eindhoven Eindhoven the Netherlands

**Keywords:** accountability and fairness of AI, artificial intelligence, clinical decision‐making, eating disorders, large language models, machine learning, psychiatry

## Abstract

Artificial Intelligence (AI) is being rapidly integrated into healthcare, but Linardon et al. reveal a troubling gap between what AI actually is, its capabilities, and the patients' and clinicians' perceptions of it—equating AI solely with large language models. In this commentary, we discuss concerns over AI's black‐box nature, its potential to perpetuate existing biases, and the blind trust some people place in its decisions, despite evidence that quantitative models outperform large language models in clinical decision‐making tasks. While AI holds promise in eating disorder care, its integration requires a nuanced understanding of its capabilities, limitations, and the critical distinction between AI for administrative automation, clinical decision‐making, and direct‐to‐patient AI. Poorly designed AI alerts risk becoming just another ignorable nuisance, while patient‐facing AI could either empower individuals or drown them in notifications and misinformation. Before we anoint AI as healthcare's savior, it requires validation for accuracy, reliability, fairness, real‐world usability, and its actual measurable impact on clinicians and patients. The real challenge is not whether AI will change healthcare but ensuring it does so responsibly—by integrating it thoughtfully into workflows, such that it is supporting rather than replacing clinical judgment, and maintaining accountability when things go wrong.

With Artificial Intelligence (AI) now more accessible than ever—thanks to popular large language models like ChatGPT and Google Bard (currently known as, Gemini)—it is being eagerly injected into every anthropocentric sector, healthcare included. But when it comes to human lives, a bit more caution is warranted. Understanding which healthcare challenges AI can genuinely address—and how—is crucial for healthcare professionals, patients, and policymakers. Linardon et al. (2025) explored this from the perspective of end‐users: individuals experiencing eating disorder (ED) behaviors (hereafter referred to as “patients”, even though it might include individuals whose symptoms are less severe than those formally diagnosed with EDs) and the clinicians treating them. Their findings highlight the prominent gap between what AI is—and can realistically do—and what these groups expect from it. The study also suggests that the disparity in opinions about AI within each demographic is closely tied to individuals' technological savviness.

Compounding this gap between AI's realistic capabilities and user expectations is the broad and often subjective perception of what actually constitutes AI. Depending on who you ask, AI could mean anything from rule‐based models crafted from domain expertise to logistic regression (which the statistics community may not be likely to accept kindly) to the more complex, non‐linear models with transformer‐based architectures (Caruccio et al. 2024). Unfortunately, it appears that the definition and examples of AI provided by Linardon et al. (2025) did little to clarify this for the study demographics. This ambiguity, coupled with the prior (in)experience of the study demographics with ‘AI’, likely contributed to a common misconception among both groups: that large language models represent the entirety of AI. This assumption is not just an oversimplification, it is a potentially hazardous one, especially when AI is entrusted with decision‐making in clinical contexts. Before delving into why this assumption is worrying, let us dissect the description of AI that was provided in Linardon et al. (2025), and clarify certain terms and concepts.

## 1 Describing Artificial Intelligence (AI)

The description of AI provided in Linardon et al. (2025) begins with: “AI is focused on creating the systems that can perform tasks that require human‐intelligence…” This is not entirely accurate. While recent models—especially in generative AI (GenAI) – can generate code to build such systems, and even verify them using automated reasoning, GenAI does not define the whole of AI. This definition also overlooks an important dimension: scalability. As the UK's National Health Services (NHS) wryly notes, even a pocket calculator performs tasks that once required human intelligence. If scalability is ignored, should we then declare the calculator an early AI success? Similarly, take the Oxford Mental Illness and Suicide (OxMIS) risk calculator, designed to estimate suicide risk in individuals with severe mental illness. While it offers a precise‐looking figure (say, a 0.7% risk over 12 months), it is not an AI model. OxMIS is built on logistic regression which is, in this case, a statistical model and not machine learning (see Ghosh et al. (2024) for an explanation of the difference). So, even if the output is dressed up with sleek probabilities, what is working underneath is traditional statistics, not AI. It models relationships already suspected by clinicians, guided by domain knowledge and existing data. Google's definition of AI offers a more useful distinction: ‘Artificial intelligence is a field of science concerned with building computers and machines that can reason, learn, and act in such a way that would normally require human intelligence or that involves data whose scale exceeds what humans can analyze.’ Now we are talking.

Machine learning models are a subset of AI, typically divided into deep learning and so‐called ‘traditional’ or shallow ML. Deep learning includes convolutional neural networks, useful for analyzing medical images (e.g., identifying biomarkers from magnetic resonance imaging or computed tomography), and recurrent neural networks, which handle sequential data like time series (think: staff scheduling, or early relapse detection), signal processing (e.g., electroencephalography‐based biomarkers), and natural language processing. Large language models sit at the intersection of advanced natural language processing and GenAI. Unlike predictive models, GenAI focuses on content generation. However, large language models can still support diagnostic or prognostic tasks—for example, by extracting diagnostic codes and clinical values from clinician's notes, and structuring them into structured tabular formats. But when it comes to clinical decision support, it is the quantitative computational models (i.e., non‐text‐based machine learning and deep learning models) that shine—predicting diagnoses, prognoses, risk of readmission or relapse, or even identifying triggers and “glimmers” in patient‐logged self‐monitoring data. And let us not forget recommendation systems, another ML flavor, helping clinicians and patients with EDs personalize treatment plans. Previous research has highlighted the risks: when large language models and natural language processing models were compared to quantitative predictive models (both expert‐defined and data‐driven), the results measured by accuracy, F1‐score, and sensitivity were, let us say, less than reassuring (Caruccio et al. 2024).

So, AI is essentially an umbrella term that conveniently shelters an eclectic mix of methodologies—from rigidly logical rule‐followers to wildly non‐linear stochastic models—each stubbornly claiming the title of “intelligence.” Attempting to pin down one universal definition feels like trying to neatly organize a group therapy session filled with clinicians who passionately disagree about nearly everything: amusing, insightful, but ultimately futile.

## 2 Perspectives on AI by the Study Demographics: Our Concerns

Given that 59% of clinicians and (only) 18% of patients reported prior experience with AI—experience mainly limited to GPT‐4‐powered large language models (e.g., ChatGPT, Copilot, Gemini), whose workings are about as transparent as a secret family recipe—it is hardly surprising that neither group seems well‐acquainted with “real” quantitative AI models. Of course, large language models are the latest trend, but there is a difference between “giving something a try” and truly understanding how it operates. Unsurprisingly, Linardon et al. (2025) revealed some interesting insights: (a) only 28% of clinicians and 11% of patients disagreed that AI—read: text‐based models—can improve accuracy; (b) only 18% of clinicians and 24% of the patients disagreed that AI can magically eliminate healthcare disparities; and (c) only 25% of clinicians and 16% of the patients expressed doubts about AI's ability to guarantee anonymity and privacy, while 45% of clinicians and 64% of the patients put their faith in these “incomparably secure” systems—even though there is no requirement for the general‐purpose large language models to comply with HIPAA or GDPR in practice.

Furthermore, as long as the model's inner workings remain off‐limits, the possibility remains high that these biases will do more to exacerbate inequalities than to reduce them (Obermeyer et al. 2019). Evaluating the fairness, reliability, and trustworthiness of AI models becomes difficult when they are black‐box, impacting accountability—especially when a decision made by a deployed AI model, trusted by a clinician, results in an undesired outcome. Although 61% of clinicians and 71% of patients reported moderate to strong concerns about the black‐box nature of AI, their actual hands‐on experience with AI was primarily with advanced search and autocomplete tools. This obscures the realization for both demographics that they may already be using and trusting such black‐box models in their daily lives. AI models, especially the larger and more complex ones, are trained on vast reserves of retrospective data. As a result, they are likely to learn and perpetuate the harmful biases present in these datasets. The more advanced models, such as some versions of widely accepted large language models, have even adopted the human flaw of hallucination (a problem experts are actively trying to address), evidenced by the disbarment of two New York lawyers in 2023 after relying on large language model’ hallucination‐generated legal references (Obermeyer et al. 2019). Relying on models that might have these harmful biases is hardly compatible with the idea of “accessible mental healthcare for everyone”—especially when an opaque algorithm quietly decides who receives help, while everyone stays cheerily focused on high performance (Obermeyer et al. 2019) and seemingly impeccable privacy.

Despite the prevailing cynicism surrounding AI, it holds promise in ED care—whether in the early detection of at‐risk individuals, predicting relapse with greater accuracy, or personalizing treatment approaches (Ghosh et al. 2024). When discussing AI's integration into the ED patient care pathway, it is important to distinguish between its various roles, because in our opinion it comes with different ethical, legal, and practical considerations, such as technical feasibility or clinician and patient acceptance—considerations that vary greatly depending on whether AI is used for administrative tasks, clinical decision‐making, and being directly accessible to patients. This differentiation necessitates a clear understanding of the different types of AI, their capabilities and limitations, and perhaps most importantly, where in a clinician's workflow each type of AI model is actually useful. Equally essential is the recognition that any AI‐generated input capable of influencing a patient's behavior—be it eating patterns, self‐awareness, or physical activity—should be considered an intervention. In the following sections, we categorize the findings of Linardon et al. (2025) into this framework and share our complementary perspectives as a psychiatry resident and an AI researcher.

### 2.1 AI for Administrative Tasks

In administrative tasks, AI could significantly boost operational efficiency, thereby reducing clinician burden and burnout, and thereby shorten those dreaded patient waitlists. AI does so by automating essential but time‐consuming processes such as appointment scheduling, insurance verification, waitlist management (e.g., prioritizing patients based on clinical urgency), patient follow‐up (e.g., predicting no shows, detecting missed sessions), and staffing and facility resource management (e.g., predicting length of hospital stays or future admission rates, managing bed availability, and predicting outpatient group session attendance). AI, specifically large language models, can even generate summaries of patient‐clinician conversations, which allows clinicians to be more ‘in the moment’ as they do not have to concern themselves with also jotting down the specifics of the conversation. However, the efficiency and reliability of large language models in transcribing and summarizing these conversations depend significantly on the language used. When patient‐clinician conversations occur in languages other than English, such as Dutch, Greek, or Tamil, relying on general‐purpose large language models might not be the best idea. This limitation becomes even more pronounced when bilingual patients use an amalgamation of two languages during a conversation. Thankfully, collaborative research initiatives between AI and clinical teams are trying to address this language problem by fine‐tuning foundation models to the specific medical domain and local languages. Nevertheless, it is rather perplexing, if not downright ironic, to observe a lack of enthusiasm for harnessing AI for these low‐risk administrative tasks, which most models might be better equipped to handle than the average overworked clinician.

### 2.2 AI for Clinical Decision‐Making

AI for clinical decision‐making in ED care has demonstrated promising potential (Ghosh et al. 2024). Yet, to move beyond the role of cryptic Oracles issuing numerical risk scores, they must be embedded in structured decision‐support pathways. For instance, rather than merely announcing that a patient's relapse risk has soared to ominous heights, such a system could automatically trigger additional psychological evaluations or expedite relevant laboratory analyses. Pairing predictions with confidence intervals further empowers clinicians to apply professional judgment, rather than deferring blindly to algorithmic outputs. Of course, dazzling explanations mean little without robust accuracy. It is critical that AI tools are validated in ED‐specific populations—not just broad “mental health” cohorts—to reflect the unique complexities of these patients. For instance, frequent exercise might be praised as a sign of a healthy, active lifestyle in general mental healthcare or primary care settings. However, in anorexia nervosa, it often serves as a compensatory mechanism driven by pathological fears of weight gain and/or a compulsive symptom, where movement becomes an uncontrollable urge, leading to significant distress when interrupted.

Additionally, explainable AI approaches aim to demystify the notorious “black‐box” nature of many AI models by exposing the key factors driving a system's decision. This, in turn, enables scrutiny of fairness, transparency, and potential bias embedded in these modern‐day Oracles. For example, if an explainable AI tool shows that a decision was made based on changes in a patient's BMI, the presence of comorbidities, and a preponderance of key patient‐reported symptoms, then the decision is likely to be fair. This contrasts with a scenario where the decision is based on the healthcare costs incurred by the patient, and the correlation of these costs with their health risks (Obermeyer et al. 2019). While healthcare costs and health risks (and thus the need for care) are highly correlated, it is a naive and precipitous assumption that all those who needed care actually received it. In short, the now‐clichéd but ever‐true dictum: correlation does not imply causation. Obermeyer et al. (2019) also raise a broader cautionary note regarding the use of high‐performing outcome prediction models for clinical decision support—especially when applied to treatment selection. Most outcome prediction models are not causally informed: they predict outcomes based on historical patterns without accounting for the effect of the intervention itself on the outcome. In practice, this can create self‐fulfilling dynamics, where the model influences the very outcome it aims to predict.

### 2.3 Direct‐To‐Patient AI

AI‐driven tools—ranging from chatbots and self‐monitoring apps to guided modules—show considerable promise in expanding 24/7 access to resources for patients with EDs. However, as any guided module constitutes an intervention, suggestions made by AI companions must be rigorously validated to ensure they are always appropriate for the patient. Clinical and AI researchers alike must safeguard against well‐intended but potentially harmful recommendations that could, in practice, do more harm than good. In 2023, an AI chatbot was suspended for giving harmful advice related to EDs, demonstrating that despite good intentions and even validation through randomized controlled trials, flaws within AI models can still occur.

Linardon et al. (2025) especially highlight the risks of ED‐supporting AI companions that provide feedback based solely on calorie count, an approach that, while seemingly objective, can dangerously reinforce disordered behaviors. The problem becomes even more pronounced when patients naively equate such easily accessible AI‐driven models to their human clinicians—a misunderstanding with consequences that, to mildly put it, are not even remotely comparable to that of asking a large language model about the rules of Squash in the absence of a referee. To maximize the usability and safety of such tools, these self‐help platforms should provide step‐by‐step instructions, transparent symptom trackers, and automated prompts advising patients when to contact a clinician. Additionally, they could simplify their clinician‐suggested reading materials and send in‐app alerts reminding them to employ coping strategies prescribed by their human clinician—ensuring that AI serves as an extension of clinical care rather than an unsupervised substitute.

## 3 Moving Forward

Before we crown AI as the panacea for healthcare's inefficiencies, a few practicalities demand attention. Algorithms must be validated—not only for performance, fairness, and reliability, but also for their real‐world impact on clinician and patient satisfaction, healthcare costs, patient access, and outcomes. Poorly timed or rigid AI‐generated alerts lacking clinical context often do little more than sharpen the clinician's instinct to override them (Vasey et al. 2021). To truly support care, AI must be thoughtfully integrated into workflows, with user‐friendly interfaces and clear professional guidelines. Meanwhile, patient‐facing AI tools bring additional challenges, from privacy concerns to the inconsistent quality of consumer apps. These tools might empower individuals with real‐time health insights—or drown them in dubious advice. Both clinicians and patients need training in responsible AI use, with the understanding that to err is human, and AI. Red flags should be reported, just as they would be with a human clinician. Also, if it is the sass of large language models that engages patients and clinicians, then let us build systems where the large language model is the friendly interface, but the actual recommendation comes from explainable, trustworthy machine learning models—as suggested by Caruccio et al. (2024).

**FIGURE 1:**
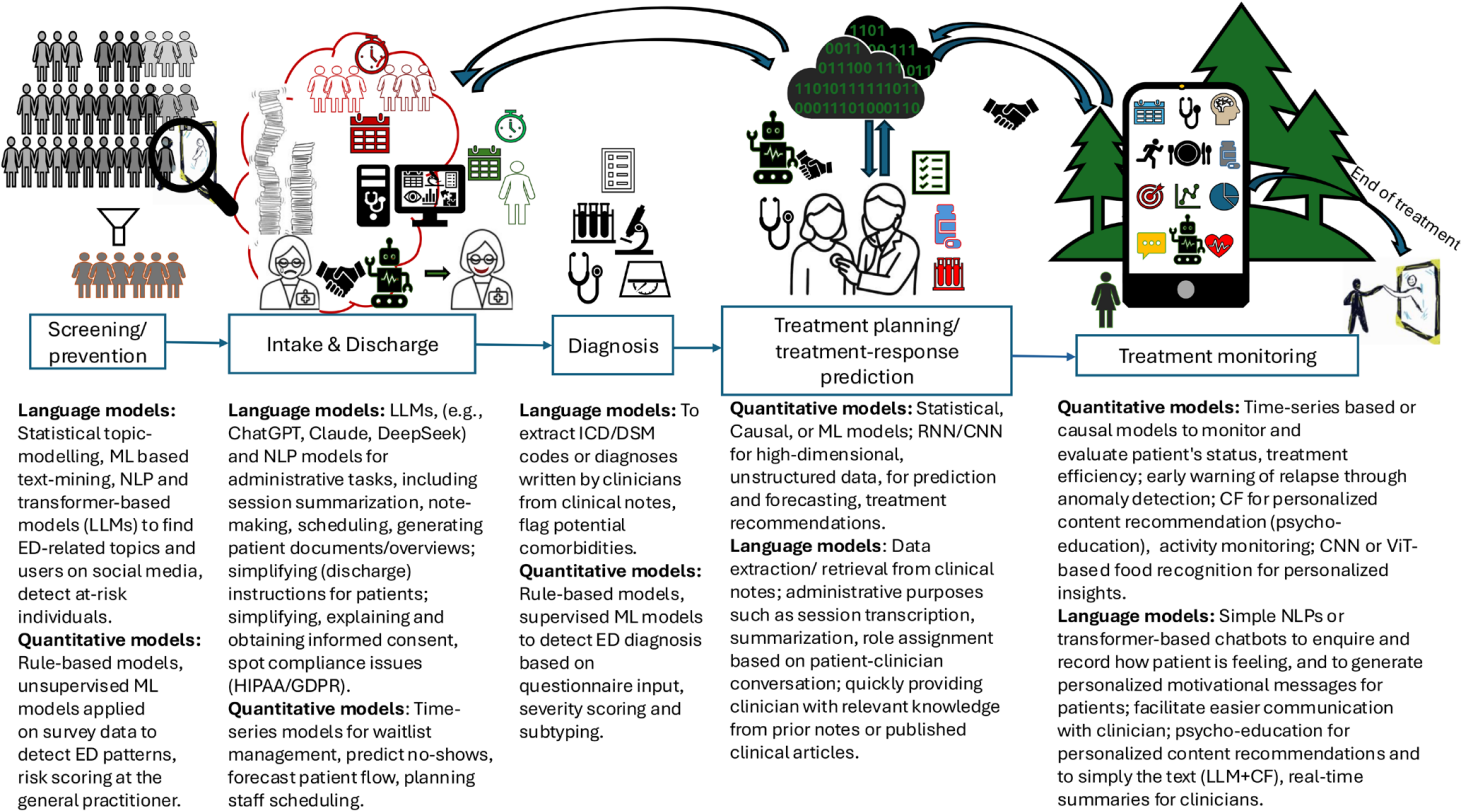
Patient care pathway, along with a non‐exhaustive list of examples illustrating how language or quantitative models could enhance administrative tasks, clinical decision‐making, or direct‐to‐patients interactions. Abbreviations: LLMs, Large language models; ML, Machine learning; NLP, Natural language processing; GPT, Generative pre‐training transformer; NN, Neural network; RNN, Recurrent NN; CNN, Convolutional NN; CF, Collaborative filtering; ViT, Vision Transformer.

Ultimately, the real question is not whether AI will change ED healthcare, but how and in which points of the workflow and the patient journeys it can do so responsibly. Some (non‐exhaustive) suggestions are made in Figure 1. If implemented thoughtfully, AI could streamline workflows, enhance clinical decision‐making, allow for clinical knowledge discovery, and even empower patients. If not, well—at least the paperwork will still get done, albeit by a digital assistant instead of a weary clinician, hopefully.

## Author Contributions

**Pia Burger:** writing – original draft, writing – review and editing, conceptualization, visualization – preparation and creation. **Sreejita Ghosh:** conceptualization, writing – original draft, writing – review and editing, visualization – preparation and creation.

## Conflicts of Interest

The authors declare no conflicts of interest.

## Data Availability

Data sharing not applicable to this article as no datasets were generated or analysed during the current study.
